# Oxidative Stress Upregulates the Transcription of Genes Involved in Thiamine Metabolism

**DOI:** 10.3906/biy-1801-51

**Published:** 2018-10-25

**Authors:** Burcu KARTAL, Ahmet AKÇAY, Bedia PALABIYIK

**Affiliations:** 1 Department of Genetics and Bioengineering, Faculty of Engineering, Alanya Alaaddin Keykubat University , Antalya , Turkey; 2 Department of Molecular Biology and Genetics, Faculty of Science, İstanbul University , İstanbul , Turkey; 3 Department of Molecular Biology and Genetics, Institute of Graduate Studies in Science and Engineering, İstanbul University , İstanbul , Turkey

**Keywords:** Fission yeast, thiamine, oxidative stress, glucose metabolism

## Abstract

Thiamine is a major vitamin that acts as a cofactor in energy metabolism in all organisms, as well as in lipid and amino acid metabolisms, and is associated with many diseases. It is known that glucose starvation decreases the intracellular thiamine pool while increasing oxidative stress tolerance. Earlier, in whole genome analysis, we detected major differences in the expression of genes related to thiamine pathway against oxidative stress in Schizosaccharomyces pombe. We investigated the effects of oxidative stress and glucose repression to thiamine pathway in S. pombe by comparing some genes encoding key enzymes of each related pathway at the transcription level. In the present study, we found that the expression of genes related to thiamine biosynthesis and transport (thi2, thi3, and pho1) increased in wild type and ird11 cells grown in thiamine-rich media under oxidative stress induced by H2O2. Based on our findings, we suggested that there might be an important effect of oxidative stress on thiamine biosynthesis and transport.

## 1. Introduction

Thiamine diphosphate, the biological active form of thiamine (vitamin B1), is a cofactor for many enzymes (pyruvate dehydrogenase, α-ketoglutarate dehydrogenase, α-ketoacid dehydrogenase, transketolase, and pyruvate decarboxylase) in universal metabolic pathways such as glycolysis, pentose phosphate pathway, and tricarboxylic acid cycle (TCA) (Friedrich, 1987) .

Yeast synthesizes 5-(2-hydroxyethyl)-4-methylthiazole phosphate (HET-P) and 4-amino-5-hydroxymethyl2-methylpyrimidine diphosphate (HMP-PP) precursors separately in thiamine biosynthesis, and the subsequent HET-P and HMP-PP unite to form ThMP with thiamine phosphate synthase (Kowalska and Kozik, 2008) . In eukaryotes, ThMP is first converted to free thiamine, and then the free thiamine is converted to ThDP (Murata, 1982) . Dephosphorylation is essential for extracellular thiamine phosphates to be transported through the cell membrane (Kowalska and Kozik, 2008) .

Various defense mechanisms (oxidative stress response) have been developed for eliminating oxidative stress that arises from the increase of reactive oxygen species that are stable within the cell. Sty1p, the stress-activated MAPK, is a major regulator in stress response (Shiozaki and Russell, 1996; Shieh et al., 1997) and triggers the global stress response via stimulating Atf1p and Pap1p transcription factors. Activated transcription factors stimulate the expression of genes that encode oxidative stress response proteins such as glutathione peroxidase (Gpx1), neutral trehalose (Ntp1), cytoplasmic catalase (Ctt1), thioredoxin reductase (Trr1), and superoxide dismutase (Sod1) (Toone et al., 1998; Mutoh et al., 2002) .

Using microarray technology earlier, we found global changes in the gene expressions in response to oxidative stress induced by H2O2 in wild type and ird11 mutant (resistant to glucose repression and oxidative stress) cells grown in optimal conditions (Kig et al., 2005; Palabiyik et al., 2012) . Because of the involvement of some of these genes in thiamine metabolism (Palabiyik et al., 2014) , we aimed to investigate the relationship between oxidative stress, glucose metabolism, and thiamine metabolism. Expression levels of thiamine metabolism, glucose metabolism, and stress response pathways genes were measured with quantitative real-time PCR (qRT-PCR) at a level of transcription, in both normal and hydrogen peroxide-induced oxidative stress conditions with/without thiamine.

## 2. Materials and methods

### 2.1. Growth conditions and S. pombe strains

S. pombe Lindner liquefaciens (wild type, 972h-) strain and ird11 mutant (Kig et al., 2005) were cultivated till midlogarithmic phase in minimal media (MML) with/without thiamine (Gutz et al., 1974). 2 mM H O was applied as an 2 2 oxidative stress agent for 1 h at 30 °C (Bayram et al., 2008) .

### 2.2. Total RNA isolation

Total RNA from control and experimental groups were acquired using a commercial kit (High Pure RNA Isolation Kit, Roche) according to the manufacturer’s recommendations. Spectrophotometrical measurements of RNA samples at 260 nm were calculated via µg/mL = A260 ´ dilution factor ´ 40 formulae (Sambrook, 1989) . RNA samples were separated into amounts of 20 μL and stored at –70 °C.

### 2.3. cDNA synthesis

cDNA synthesis from RNA molecules was done with a commercial kit (Transcriptor High Fidelity cDNA Synthesis Kit, Roche) according to the manufacturer’s instructions; cDNA samples were stored at –20 °C.

### 2.4. Real-time PCR

Variation in expression profiles of the genes of interest under different conditions was determined with quantitative RT-PCR that is based on “SYBR green”. Fast Start SYBR Green Master Kit (Roche) and “Roche, 480” were used according to the manufacturer’s instructions. Gene-specific primers were designed at Primer 3 program and are listed in Table. S. pombe actin gene (act1) was used as a reference gene for normalization of the results (Xue-Franzen et al., 2006) .

**Table:** Primers used throughout the study. (¹: Forward sequence; ²: Reverse sequence).

Group	SEQ_ID	Name	Primer sequence (5’-3’)
Reference gene	SPBC32H8.12c	Actin / act1	AGATTCTCATGGAGCGTGGT¹ TCAAAGTCCAAAGCGACGTA²
Thiamine metabolism	SPAC17A2.01	High-affinity import carrier for pyridoxine, pyridoxal, and pyridoxamine / bsu1	GCCCGTTTACTTTTGTTCCA¹ GCAACACCGATGATGAAATG²
	SPAC23H4.10c	Bifunctional thiamine-phosphate dipyrophosphorylase/hydroxyethylthiazole kinase / thi4	GTGATGGGTGTAACGGCTTC¹ GAGTTTTTCGCTTCCACTGC²
	SPBC26H8.01	Thiazole biosynthetic enzyme / thi2	CCCATTTGGTTGTTTCTGCT¹ CGCATGTCGTGAAGGTTAGA²
	SPBP4G3.02	Acid phosphatase / pho1	AGCATTGACTTTCCCACCAC¹ ATTCCAACAGCATCGAAAGC²
	SPBP8B7.18c	Phosphomethylpyrimidine kinase (predicted)	GCAGCCCTGAAATCGTTAAG¹ CGAGAGAATCCCCAGAAGTG²
	SPCC18B5.05	Phosphomethylpyrimidine kinase (predicted)	GACGGCCGATCTGATTTATG¹ TGGCAGCTGTAAGAGAGCAA²
	SPCC1223.02	4-amino-5-hydroxymethyl-2-methylpyrimidine phosphate synthase / nmt1	TCCCCAGAGATTGGAACAAG¹ GGTCAAGTTCCCAGGTCAAG²
Glucose metabolism	SPBC1198.14c	Fructose-1,6-bisphosphatase / fbp1	GTATGGTGCTTCGGCTCATT¹ TTCATGTTTCGATGGGTCAA²
	SPAC4F8.07c	Hexokinase 2 / hxk2	CAACAAGGACTTTGCCCAAT¹ AAGGTGTCGCTCTCCTTTGA²
Stress response	SPAC24B11.06c	MAP kinase / sty1	TGTTCATTCTGCCGGTGTTA¹ GAATACGAGCCAAACCGAAA²
	SPAC821.10c	Superoxide dismutase / sod1	ATTGGCCGTACCATTGTCAT¹ GACACCACAAGCGTTACGTG²
	SPCC757.07c	Catalase / ctt1	ATCCTCAATCCGACCACTTG¹ AACGTCGGTAATTTCGTCCA²

PCR was carried out under the following conditions: 95 °C for 10 min (preincubation), followed by 45 cycles of 95 °C for 10 s, 53 °C for 7 s, and 72 °C for 5 s. 2.5. Statistical analysis The variation in gene expression level (R) was calculated according to Pfaffl equation (Pfaffl 2001 ), which is based on the proportion of crossing threshold (Ct) measured for target and reference genes depending on the yield at each reaction (E). Statistical analysis of experiment results was done with “Graphpad Ver.5” software.

R = (E_target gene_) ^ΔCttarget gene(control-test)^ / (E_reference gene_) ^ΔCtreference gene(control-test)^

## 3. Results

### 3.1. The effect of thiamine on the expression of genes related to thiamine metabolism

**Figure 1:**
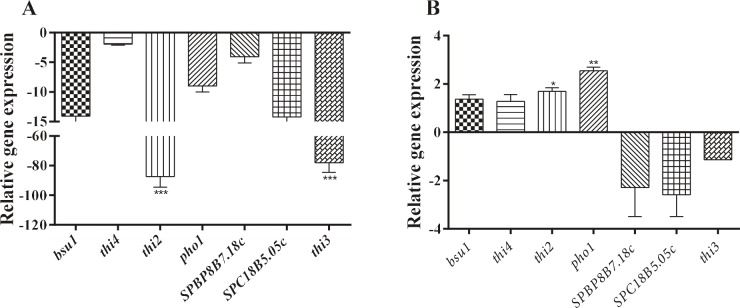
Relative expression profiles of genes related to thiamine metabolism. The expression of genes related to thiamine pathway in S. pombe cells growing in thiamine-rich media calculated relative to thiamine-free media. A. S. pombe wild type; B. S. pombe ird11 mutant strain. Statistical analysis of experiment results was done with “Graphpad Ver.5” software. Error bars represent standard deviation of three experimental replicates. (Dunnet’s test, P < 0.05*, P < 0.01**, P < 0.001***).

Expression profiles of genes that are included in thiamine intake and biosynthesis pathway were examined in S. pombe 972h- wild type and ird11 mutant strains grown in thiamine-rich media (Figure 1). The expression of thi2 and thi3 genes was downregulated (87.29´, 78.06´, respectively) in the wild type, as expected (Figure 1A), while in ird11 strain the expression of thi3 did not change, but thi2 and pho1 genes were upregulated (1.69´, 2.55´, respectively) (Figure 1B).

### 3.2. Thiamine-mediated glucose metabolism and stress response pathway

**Figure 2:**
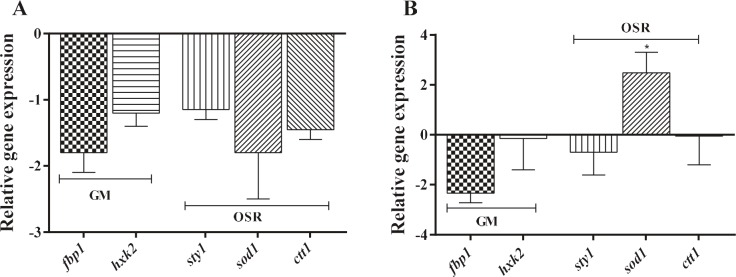
Relative expression profiles of genes related to glucose metabolism and oxidative stress response pathways. Expression of genes of interest in S. pombe cells growing in thiamine-rich media was calculated relative to thiamine-free media. A. S. pombe wild type; B. S. pombe ird11 mutant strain. Statistical analysis of experiment results was done with “Graphpad Ver.5” software. Error bars represent standard deviation of at least two experimental replicates. (Dunnet’s test, P < 0.05*). GM: glucose metabolism; OSR: oxidative stress response.

The expression profiles of genes involved in glucose metabolism and stress response pathways were examined in S. pombe 972h- wild type and ird11 mutant strains grown in thiamine-rich media, using qRT-PCR (Figure 2). It was determined that the pfb1 gene encoding phosphofructokinase 1 enzyme, which is a gluconeogenetic enzyme, was downregulated, although not statistically significantly in both of the strains at the presence of thiamine. No significant variation was observed in the expression of the hxk2 gene encoding hexokinase 2 enzyme, which starts glycolytic flow. It is suggested that there is an optimal glycolytic flow in both of the strains in all growth media. The transcription of stress response genes (sty1, sod1, and ctt1) did not change in both strains in the absence and presence of thiamine (Figures 2A and 2B). Only the expression of the sod1 gene, which encodes the superoxide dismutase 1 enzyme in ird11, was increased (2.48´) in thiamine-rich media relative to thiamine-free media (Figure 2B).

### 3.3. Activation of thiamine metabolism related genes under oxidative stress

**Figure 3:**
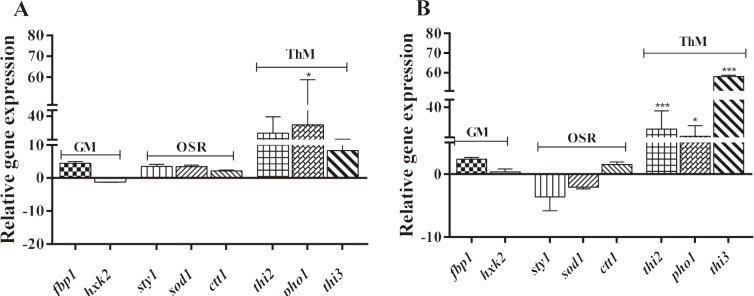
Relative expression profiles of genes related to thiamine metabolism, glucose metabolism, and oxidative stress response pathways under oxidative stress. After S. pombe cells grown until midlogarithmic phase were exposed to 2 mM hydrogen peroxide, inducing oxidative stress in presence of thiamine, expression profiles of the genes were calculated relative to stressfree condition. A. S. pombe wild type; B. S. pombe ird11 mutant strain. Statistical analysis of experiment results was done with “Graphpad Ver.5” software. Error bars represent standard deviation of at least three experimental replicates. (Dunnet’s test, P < 0.05*, P < 0.001***). GM: glucose metabolism; OSR: oxidative stress response; ThM: thiamine metabolism.

Expression levels of genes encoding main enzymes involved in oxidative stress response pathways, thiamine metabolism, and glucose metabolism were determined at the level of transcription after exposing S. pombe wild type and ird11 cells to oxidative stress (2 mM H2O2, 1 h) in thiamine-rich media (Figure 3). No significance was observed in the expression of genes related to glucose metabolism and oxidative stress response pathway in both strains. In wild type, a significant increase was observed only in the expression of the pho1 gene encoding acid phosphatase, which allows thiamine to be introduced into the cell (Figure 3A). On the other hand, the expression of the thi2 and thi3 genes, which encode thiazole biosynthetic enzyme (participates in the synthesis of the thiazole ring) and 4-amino-5-hydroxymethyl-2-methylpyrimidine phosphate synthase enzyme (participates in the synthesis of the pyrimidine ring), respectively, and also the expression of pho1 gene were upregulated in ird11 as if thiamine was absent in the media (Figure 3B).

## 4. Discussion

There are many critical diseases occurring due to thiamine deficiency with little progress in diagnosis and treatment. Thiamine deficiency increases oxidative stress in neurodegenerative diseases such as Alzheimer, Parkinson, Huntington, and Wernicke-Korsakof syndrome; furthermore, thiamine-dependent enzymes are more sensitive to oxidative stress (Gibson et al., 1999; Lin and Beal, 2006; Hazell et al., 2010; Jhala and Hazell, 2011) . It is also known that thiamine has an important role in signal transduction, immune system activation, and signaling in animal cells (Manzetti et al., 2014) . It is believed that determining the expression profiles of genes responsible for thiamine biosynthesis and transport might lead to research into the diagnosis and treatment of the diseases. In this study, we investigated the relationship between thiamine metabolism, oxidative stress response, and glucose metabolism in both S. pombe wild type and the ird11 mutant (Kig et al., 2005; Palabiyik et al., 2012) .

The expression of genes related to biosynthesis and transport of thiamine is completely suppressed by thiamine in the media, whereas in the absence of thiamine it is known that their expression increases at a high level (Maundrell, 1990; Praekelt et al., 1994; Nosaka, 2006) .

Besides, it has been reported that in S. pombe, thiamine represses the mRNA synthesis of many genes that are involved in their own metabolism, such as thi2, thi3, thi4, pho4, and car1 (Schweingruber et al., 1991) . It has been shown that with microarray analysis, in the absence of thiamine in S. cerevisiae, the expression of THI5, THI12 (ortholog of S. pombe thi3 gene), and THI4 (ortholog of S. pombe thi2 gene) genes were significantly increased (No saka et al., 2005). It was confirmed in the present study by the remarkable decrease (approximately 80-fold) in the expression of thi3 and thi2 genes in S. pombe wild type cells growing in the presence of thiamine, compared to the expression of other genes associated with thiamine biosynthesis and transport (Figure 1A).

In the presence of thiamine, the fact that the pfb1 gene in both strains was downregulated suggested continuing glucose repression (Figures 2A and 2B). Likewise, the fact that no significant decrease was observed in stress response genes expression (sty1, sod1, and ctt1) in the wild type under thiamine-rich conditions (Figure 2A) suggests that the stress response is not effect ed by environmental thiamine. However, differences in the expression profiles of genes related to thiamine and oxidative stress response pathways between ird11 and wild type cells might result from a lack of glucose repression in ird11 (Figures 1B and 2B).

Palabiyik et al. (2014) have shown the relationship between oxidative stress and thiamine metabolism in microarray analyses, indicating that the expression of the thi3 gene is 33.5-fold less than wild type in ird11, but 31-fold higher in ird11 exposed to H2O2. We determined an in creased expression of genes involved in thiamine biosynthesis and transport (thi2, thi3, and pho1) when ird11 and wild type cells were exposed to H2O2 (Figure 3). Therefore, it is suggested that S. pombe cells need thiamine in defense mechanisms developed against oxidative stress. Recently, it has been reported that osmotic and oxidative stresses elevate the transcription of thiamine biosynthesis genes in oil palm (Elaeis guineensis) (Yee et al., 2016; Idris et al., 2018) .

Consequently, the fact that expression of genes related to thiamine biosynthesis and transport (thi2, thi3, and pho1) increased when wild type cells were exposed to H2O2 (Figure 3A), while the expression of these genes decreased under thiamine-rich conditions (Figure 1A) in this study, suggests that oxidative stress upregulates thiamine metabolism to protect the cellular balance in fission yeast. This conclusion is supported by the findings obtained from ird11, which is resistant to glucose repression and oxidative stress (Figures 1B and 3B). Also, it has been reported that thiamine is involved in the stabilization of the redox levels of cells throughout the production of NAPDH and glutathione during oxidative stress response and protects the tissues against oxidative damage via reduced NADP+ (Depeint et al., 2006; Gioda et al., 2010) . Besides, it has been put forward that in S. cerevisiae cells, free radical levels and protein oxidation have decreased under different stress conditions in the presence of thiamine (Wolak et al., 2014) . Based on our findings, we hypothesize that thiamine pathway is affected by oxidative stress.

## Acknowledgments

This research was funded by the Scientific Research Project Coordination Unit of İstanbul University, project ID: 28781, and by The Scientific and Technological Research Council of Turkey, project number: 1919B011403361.
